# Short-term moderate caloric restriction in a high-fat diet alleviates obesity via AMPK/SIRT1 signaling in white adipocytes and liver

**DOI:** 10.29219/fnr.v66.7909

**Published:** 2022-05-03

**Authors:** Shaohong Zhang, Shuoshuo Sun, Xiao Wei, Mengxiao Zhang, Yu Chen, Xiaodong Mao, Guofang Chen, Chao Liu

**Affiliations:** 1 Endocrinology Department, Affiliated Hospital of Integrated Traditional Chinese and Western Medicine, Nanjing University of Chinese Medicine, Nanjing, China; 2 Department of Geriatrics, The Affiliated Huaian No. 1 People’s Hospital, Nanjing Medical University, Nanjing, China; 3 Department of Geriatrics, Yancheng TCM Hospital Affiliated to Nanjing University of Chinese Medicine, Yancheng, China

**Keywords:** obesity, calorie restriction, high-fat diet, AMPK/SIRT1 pathway

## Abstract

**Background:**

Obesity is a growing problem for public health worldwide. Calorie restriction (CR) is a safety and effective life intervention to defend against obesity. Short-term moderate CR may be a more favorable strategy against this pathology. However, the mechanisms behind the effects of CR remain to be clarified. Increased energy expenditure in the liver and brown adipose tissue could potentially be manipulated to modulate and improve metabolism in obesity. Moreover, nicotinamide adenine dinucleotide (NAD)-dependent deacetylase sirtuin-1 (SIRT1) and AMP-activated protein kinase (AMPK) are well-characterized metabolic modulators. We aim to explore the anti-obesity effects of short-term moderate CR by improving energy metabolism via the SIRT1/AMPK pathway in white adipocytes and liver in a mouse model of obesity.

**Methods:**

Male C57BL/6 mice were randomized into two groups receiving either a standard or a high-fat diet (HFD) for 8 weeks to induce obesity. The HFD-induced obese mice were further randomized into two groups: HFD group or CR group (received 75% of the food eaten by HFD group). Their energy metabolism, white adipose tissue (WAT) contents, hepatic fat deposition, the expression of AMPK, SIRT1, peroxisome proliferators γ-activated receptor coactivator-1α (PGC-1α), nuclear factor kappa B (NF-κB), endothelial nitric oxide synthase (eNOS) in WAT, and hepatic tissues were determined.

**Results:**

After 4 weeks, body weight, total serum cholesterol, fasting blood glucose, and insulin levels were significantly lower in the CR group. Moreover, CR ameliorated hepatocyte steatosis, attenuated white adipogenesis, and increased energy expenditure and expressions of SIRT1, PGC-1α, and phosphorylated AMPK in subcutaneous WAT and the hepatic tissues. In addition, CR reduced the protein levels of NF-κB and increased the eNOS expression.

**Conclusion:**

Short-term moderate CR decreases obesity, increases the thermogenesis, and inhibits inflammation in a mouse model of obesity, probably via the activation of the AMPK/SIRT1 pathway in WAT and liver.

## Popular scientific summary

- Short-term moderate CR alleviates obesity.
- CR increases the thermogenesis in a mouse model of obesity.
- CR activates the SIRT1/AMPK pathway in white adipocytes and liver.

By inflicting 20–30% of adults worldwide, metabolic syndrome (MS) emerges as the co-occurrence of obesity, insulin resistance (IR), hypertension, and hyperlipidemia (1). Among them, obesity is a core risk factor responsible for an ever-growing list of disorders, including non-alcoholic fatty liver disease, type 2 diabetes, cardiovascular disease, chronic kidney disease, and cancers (2, 3). These disorders may all arise from a pathological accumulation of fat (4). Some anti-obesity interventions, either pharmacological or surgical, have been approved by the United States Food and Drug Administration (5). Bariatric surgeries and drugs (such as sibutramine) can reduce fat accumulation and increase energy consumption, respectively (6, 7). However, these treatments are expensive and carry other health risks, such as psychiatric disturbances. Therefore, lifestyle interventions have been proposed as a safe and effective anti-obesity strategy.

Calorie restriction (CR) is a lifestyle intervention that reduces fat accumulation and prevents hepatic steatosis (8, 9). Very low-calorie restriction (VLCR) curbs or even reverses obesity and type 2 diabetes mellitus. However, this effect cannot be maintained in the long term, and weight may be even regained. In this condition, short-term moderate CR may be a more favorable strategy. The mechanisms underlying CR-mediated metabolism remain to be clarified. The major cause of obesity is an imbalance between energy intake and expenditure. Emerging evidence suggests that modulating thermogenesis may provide a plausible means to reestablish energy balance (10). Energy expenditure in the liver and brown adipose tissue (BAT) can be manipulated to rebalance metabolism in obesity (11).

AMP-activated protein kinase (AMPK) is an ubiquitous serine/threonine protein kinase that acts as the major cellular energy sensor (12). The beneficial effects of CR involve AMPK. Activated AMPK stimulates catabolic and inhibits anabolic metabolism to restore energy homeostasis. Moreover, in metabolically sensitive tissue (including hepatic, adipose, and skeletal muscles), activated AMPK enhances the function of NAD-dependent deacetylase sirtuin-1 (SIRT1), a longevity-related factor modulating fat deposition (13–15). Peroxisome proliferators γ-activated receptor coactivator-1α (PGC-1α) is a main regulator of fatty acid β-oxidation and gluconeogenesis. Moreover, endothelial nitric oxide synthase (eNOS) and nuclear factor kappa B (NF-κB) are the common target molecules regulated by AMPK and SIRT1. The AMPK/SIRT1/PGC-1α signaling pathway acts as an energy-sensing network in energy metabolism. Whether this AMPK/SIRT1/PGC-1α pathway mediates the effect of CR on fat metabolism and thermogenesis is unknown. In addition, it is unclear whether CR may decrease obesity via NF-κB inhibition through the AMPK/SIRT1 pathway. Therefore, we explored how moderate CR induces weight loss and improves energy metabolism by modulating the AMPK/SIRT1 signaling in a murine model of obesity.

## Materials and methods

### Reagents and antibodies

Anti-AMPKα, phospho-AMPK (p-AMPKα), anti-eNOS, anti-NF-κB, β-actin, β-tublin antibodies, and horseradish peroxidase (HRP)-conjugated secondary antibodies were purchased from Wuhan Servicebio Technology Co., Ltd. (China). Anti-SIRT1 and anti-UCP1 (uncoupling protein 1) antibodies were purchased from Abcam (UK), and the anti-UCP2 (uncoupling protein 2) antibody was purchased from Cell Signalling Technology (USA). Rodent chow was supplied by the Trophic Animal Feed High-Tech Co., Ltd. (China). The compositions of high-fat chow and standard chow are shown in Table 1. Kits for the measurement of serum triglyceride (TG) and total cholesterol (TC) were purchased from Nanjing Jiancheng Bioengineering, Inc. (China). An enzyme-linked immunosorbent assay (ELISA) kit for the assessment of serum insulin was purchased from the American Laboratory Products Company (USA). The hematoxylin, oil red O, and eosin staining were purchased from Servicebio Technology Co., Ltd. (China). The radio immunoprecipitation assay (RIPA) lysis buffer and bicinchoninic acid (BCA) kit were purchased from Beyotime Biotechnology Co., Ltd. (China).

**Table 1:** Composition of the diets

Ingredient	Standard chow	High-fat diet
Casein	14.0%	19.5%
Maltodextrin	16.8%	22.5%
Corn starch	46.5%	0
Sucrose	10.0%	8.9%
Soybean oil	4.0%	3.3%
Lard	0	30.1%
Cellulose	5.0%	6.9%
Mineral	2.5%	6.8%
Vitamin	0.8%	1.4%
L-cystine	0.2%	0.3%
Choline bitartrate	0.2%	0.3%
Protein caloric ratio	14.5%	14.1%
Carbohydrate caloric ratio	76.15%	25.9%
Fat caloric ratio	9.35%	60.0%

### Animal care and handling, and experimental design

Six-week-old male C57BL/6J mice (*N* = 45) were purchased from Shanghai SLAC Laboratory Animal Co., Ltd. (China) and housed in a climate-controlled facility (22 ± 2°C) with a 12 h light–dark cycle. Following a 2-week acclimation period, the mice were randomized into two primary groups: standard diet (STD) group (*N* = 15) receiving STD (9.35% kcal from fat), while the high-fat diet (HFD) group (*N* = 30) receiving a HFD (60.00% kcal from fat). After 8 weeks, HFD mice rapidly gained weight and became obese, while STD mice remained lean throughout the experimental period. HFD mice were then randomized into two secondary groups: one group continuing to receive the HFD ad libitum, while the other group receiving 75% of the food eaten by the HFD group (high-fat diet with calorie restriction [HFDCR]). The mice had ad libitum access to water. Body weight and fasting blood glucose (FBG) were recorded weekly. After 4 weeks, all the mice were fasted for 8 h, and blood samples were taken from the orbit fossa. All the mice were euthanized with sodium pentobarbitone anesthesia (100 mg/kg, intraperitoneal). Hepatic tissue and subcutaneous white adipose tissue (sWAT) were harvested and stored at −80°C. All experimental protocols involving animals were approved by the Animal Experimentation Ethics Committee of Jiangsu Province Academy of Traditional Chinese Medicine (Nanjing, China) (approval number: AEWC-20180510-30) and in accordance with the Helsinki Declaration of 1975, as revised in 2008.

### Intraperitoneal glucose tolerance test

The mice were subjected to an intraperitoneal glucose tolerance test (IPGTT) after CR intervention. First, the mice were fasted for 8 h and weighed for calculation of glucose dosage. Then, blood samples were collected via tail vein incision to determine blood glucose prior to glucose loading. All the mice received 2 g/kg glucose via intraperitoneal injection. Finally, at 15, 30, 60, 90, and 120 min following glucose loading, blood glucose levels were measured using an automated commercial glucometer (Roche, Germany).

### Indirect calorimetry

After 4 weeks of CR, the mice were placed in a metabolic cage (TSE PhenoMaster, Germany) for 24 h to assess heat production and oxygen consumption.

### Micro-MRI scanning for abdominal WAT

After 4 weeks of CR, the mice were subjected to isoflurane gas anesthesia for 10 min to perform micro-magnetic resonance imaging (MRI) (Biospec 7T/20 USR, Bruker, Germany) scanning of the abdomen. Abdominal WAT volume in each section (thickness 2 mm) was compared between groups.

### Determination of serum indices

At the end of the study, blood samples obtained from the orbital fossa were centrifuged 1,500 × *g* at room temperature for 20 min, and then the serum was collected and stored at −80°C. Blood glucose was measured using a commercial glucometer (Roche, USA). Levels of serum TG, TC, and insulin were measured using ELISA kits, and IR was calculated using the homeostasis model assessment of IR (HOMA-IR) according to the formula: fasting insulin (mmol/L) × FBG (mIU/L) ÷ 22.5.

### Oil red O staining

To detect hepatic lipid deposition, liver tissue was fixed in optimal cutting temperature compound, sliced into 15-μm thick sections, and stained in 0.5% oil red O for 8–10 min. Thereafter, the slices were stained in hematoxylin for 3–5 min, washed in distilled water, and sealed with gelatine. Sections were examined using a light microscope (Olympus, Japan).

### Histopathology and immunohistochemistry

Adipose and hepatic tissue samples were fixed in 4% formaldehyde overnight at room temperature, paraffin-embedded, sliced into 5 μm sections, and stained using hematoxylin and eosin (H&E). Photomicrographs were acquired using a light microscope (Olympus, Japan), and quantitative analysis was performed using Image-Pro Plus 6.0 (Media Cybernetics, USA).

### Real-time quantitative reverse transcription PCR

Total RNA was extracted from sWAT and hepatic tissue using Trizol Reagent (Thermo Fisher Scientific, USA), in accordance with the manufacturer’s instructions. A Reverse Transcription Kit (Toyobo, Japan) was used to synthesize cDNA, following the manufacturer’s instructions. A Fast Real-Time PCR System using Quantstudio 7 Flex (Thermo Fisher Scientific, USA) in conjunction with a SYBR Select Master Mix Kit (Applied Biosystems, USA) was used to perform quantitative reverse transcription polymerase chain reaction (qRT-PCR), in accordance with the manufacturers’ instructions. PCR parameters were set as follows: 95°C for 1 min, then 40 cycles of 95°C for 15 s, 45°C for 15 s, and 72°C for 45 s. Primer pair sequences are shown in Table 2. The relative levels of targeted gene mRNA transcripts were quantified by comparison with the control glyceraldehyde-3-phosphate dehydrogenase (GAPDH). Relative quantitation was performed using the 2^−ΔΔCt^ method.

**Table 2:** Primer pair sequences

Gene	Forward	Reverse
*UCP1*	CAAAAACAGAAGGATTGCCGAAA	CCCAATGAACACTGCCACAC
*UCP2*	ATGGTTGGTTTCAAGGCCACA	TTGGCGGTATCCAGAGGGAA
*PGC-1*α	AGACAAGACCAGTGAACTAAGGGAT	AGGAAGAGCAAGAAGGCGACA
*GAPDH*	CCATTCTCGGCCTTGACT	TGAAGGTCGGTGTGAACG

### Western blot assay

Adipose and hepatic tissue were lysed using the RIPA buffer (50 mM Tris pH 7.4, 150 mM NaCl, 1% NP-40, 0.5% sodium deoxycholate, 0.1% sodium dodecyl sulfate (SDS), sodium orthovanadate, sodium fluoride, ethylenediaminetetraacetic acid (EDTA), and leupeptin) containing protease inhibitors. Tissue lysate was centrifuged at 12,000 × *g* for 30 min at 4°C to pellet debris, and the protein concentration of the supernatant was estimated using a BCA Kit. Proteins were separated via sodium dodecyl sulfate polyacrylamide gel electrophoresis (SDS-PAGE) and transferred to a polyvinylidene fluoride (PVDF) membrane. The membrane was incubated overnight at 4°C with primary antibodies specific to p-AMPKα (1:1,000), AMPKα (1:1,000), SIRT1 (1:1,000), PCG-1α (1:2,000), UCP1 (1:3,000), UCP2 (1:1,000), eNOS (1:1,000), NF-κB (1:1,000), β-tubulin (1:1,000), and β-actin (1:3,000), followed by incubation with secondary antibodies (1:3,000). Enhanced chemiluminescence reagents were used for detection, and images were captured via film cassette exposure (Tanon, China).

### Statistical analysis

All data are represented as mean ± standard error of the mean (SEM). All statistical analyses were performed using SPSS version 22.0. One-way analysis of variance (ANOVA) was used to compare intergroup means, followed by Dunnett’s *t*-test. Statistical significance was set at *P* < 0.05.

## Results

### CR ameliorated HFD-induced obesity

**Fig. 1:**
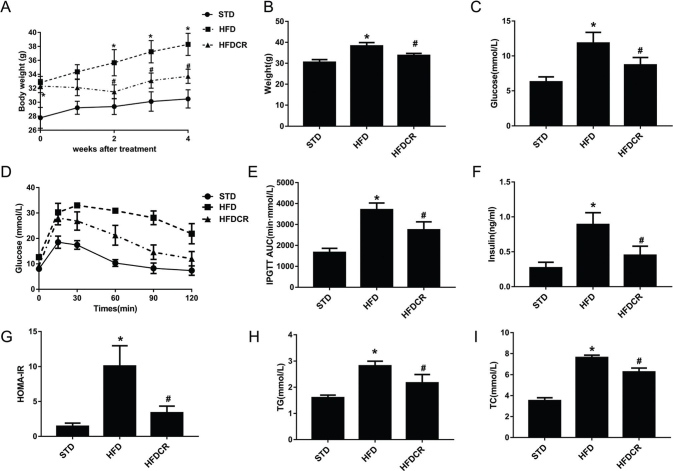
CR ameliorated high fat diet (HFD)-induced obesity and associated indicators. (a) Body weight changes after CR intervention, (b) body weights, (c) fasting blood glucose (FBG) level, (d) intraperitoneal glucose tolerance test (IPGTT) results, (e) area under the curve (AUC) of the IPGTT, (f) serum insulin level, (g) homeostasis model assessment of insulin resistance (HOMA-IR), (h) serum triglyceride (TG) level, and (i) serum total cholesterol (TC) level. Data are represented as mean ± standard error of the mean for *n* = 6–8 per group (*P < 0.05 vs. STD group and ^#^*P* < 0.05 vs*.* HFD group). Additional abbreviations: STD, standard diet; HFD, high-fat diet; HFDCR, high-fat diet with calorie restriction.

Body weight of the HFD group was 20% higher than that of the STD group during the obesity induction phase (*P* < 0.05). Body weight in the HFDCR group was significantly decreased, compared with that in the HFD group (*P* < 0.05) (Fig. 1a and b). In addition, the mice in the HFD group exhibited a significantly greater increase in FBG (*P* < 0.05), glucose intolerance levels (*P* < 0.05), insulin (*P* < 0.05), and HOMA-IR (*P* < 0.05) than those in the STD group (Fig. 1c–g), and similar trends were noted in TG (*P* < 0.05) and TC (*P* < 0.05) levels (Fig. 1h and i). Collectively, these results showed that CR countered obesity and improved glucose and lipid metabolism in HFD-fed mice.

### CR attenuated the deposition of abdominal WAT and hepatic fat

**Fig. 2:**
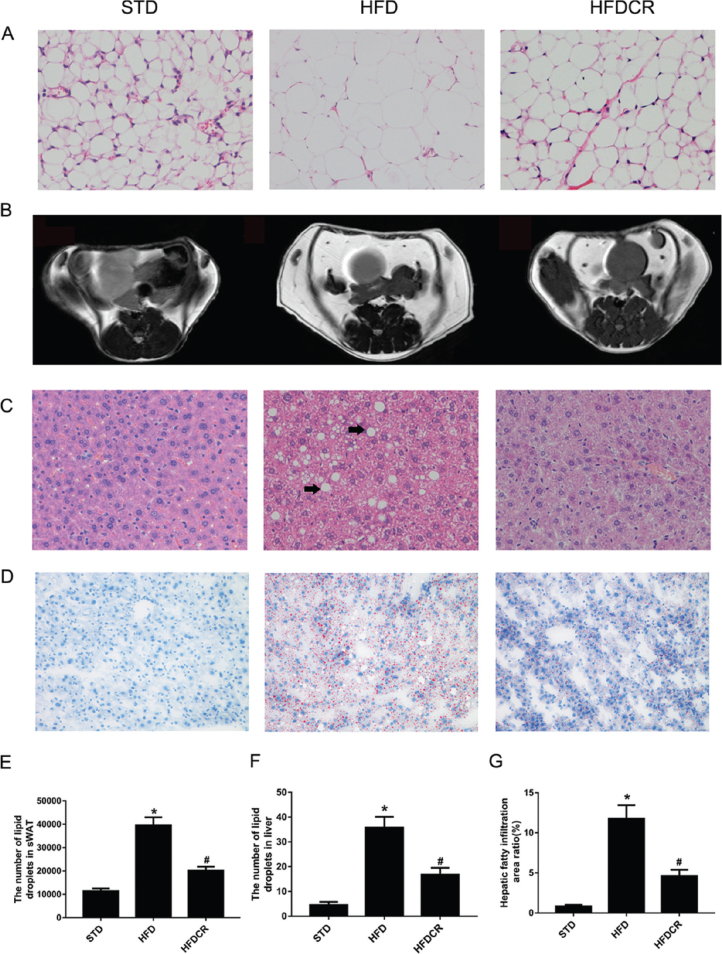
CR attenuated the deposition of abdominal white adipose tissue and hepatic fat. (a) Hematoxylin and eosin (H&E)-stained subcutaneous white adipose tissue (sWAT) (400× magnification), (b) abdominal white adipose tissue (WAT) MRI scans, (c) H&E-stained hepatic tissue (400× magnification; arrows indicate large lipid droplets), (d) oil red O-stained hepatic tissue (200× magnification), (e) sWAT adipocyte size, (f) number of hepatic lipid droplets, and (g) hepatic fatty infiltration area ratio. Data are represented as mean ± standard error of the mean for *n* = 3 per group (*P < 0.05 vs*.* STD group and ^#^*P* < 0.05 vs*.* HFD group). Additional abbreviations: STD, standard diet; HFD, high-fat diet; HFDCR, high-fat diet with calorie restriction.

The size and volume of adipocytes were obviously enlarged by HFD in the sWAT but reduced in short-term CR-treated mice (Fig. 2a). The abdominal sWAT area in the HFDCR group was smaller than that in the HFD group (Fig. 2b). Furthermore, the hepatic fat deposition in the HFD group was significantly increased (*P* < 0.05), while reduced in the HFDCR group (*P* < 0.05) (Fig. 2c and d). Semiquantitative analysis showed that the number of lipid droplets in the sWAT and liver was decreased by CR in HFD-induced obese mice as well as the hepatic fatty infiltration area ratio (Fig. 2e–g).

### CR elevated energy expenditure

**Fig. 3:**
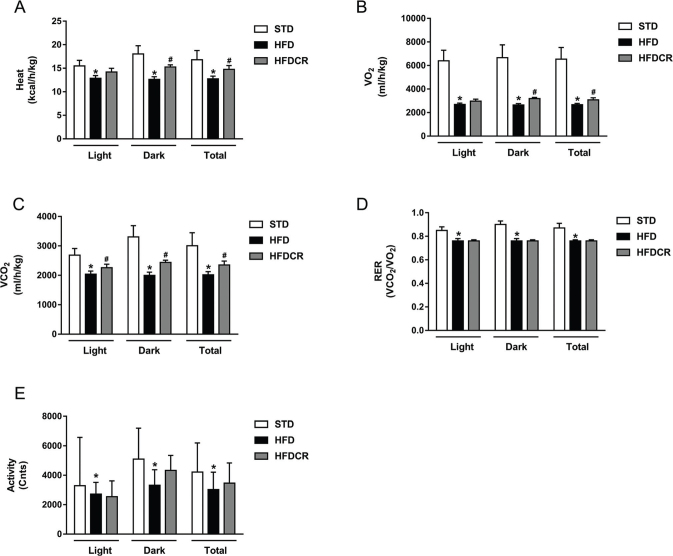
CR elevated energy expenditure. (a) Heat production, (b) oxygen consumption, (c) carbon dioxide production, (d) respiratory exchange ratio (RER), and (e) activity. Data are represented as mean ± standard error of the mean for *n* = 3–4 per group (*P < 0.05 vs*.* STD group and ^#^*P* < 0.05 vs*.* HFD group). Additional abbreviations: STD, standard diet; HFD, high-fat diet; HFDCR, high-fat diet with calorie restriction.

During the 24 h in the metabolic cage, the HFD group exhibited significantly lower heat production, oxygen consumption, carbon dioxide production, respiratory exchange ratio (RER), and ambulatory activity than the STD group (all *P* < 0.05, Fig. 3a–e). These indicators, except RER and ambulatory activity, were significantly increased by CR (*P* < 0.05).

### CR modulated the expression of thermogenesis-related genes and proteins

**Fig. 4:**
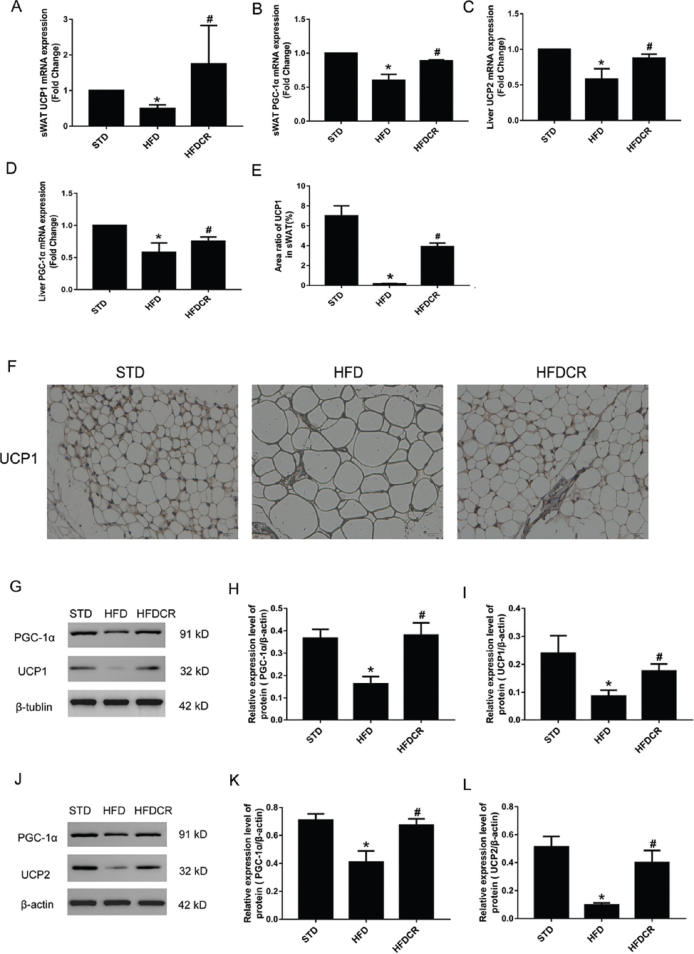
CR modulated thermogenesis-gene transcription and protein expression. (a, b) Levels of uncoupling protein 1 (UCP1) and peroxisome proliferator-activated receptor gamma coactivator 1 alpha (PGC-1α) mRNA transcripts in the subcutaneous white adipose tissue (sWAT), (c, d) levels of uncoupling protein 2 (UCP2) and PGC-1α mRNA transcripts in the hepatic tissue, (e, f) protein expression level of UCP1 in sWAT (200× magnification), (g–i) the protein levels of PGC-1α and UCP1 in the sWAT, and (j–l) the protein levels of PGC-1α and UCP2 in the hepatic tissue. Data are represented as mean ± standard error of the mean for *n* = 3 per group (*P < 0.05 vs*.* STD group and ^#^*P* < 0.05 vs*.* HFD group). Additional abbreviations: STD, standard diet; HFD, high-fat diet; HFDCR, high-fat diet with calorie restriction.

Relative transcription and protein levels of *UCP1* and *PGC-1α* in sWAT were significantly lower in the HFD group than those in the STD group (*P* < 0.05), while their mRNA and protein levels were significantly increased by CR (*P* < 0.05, Fig. 4a, b and g–i). Furthermore, mRNA and protein expressions of *UCP2* and *PGC-1α* in hepatic tissue exhibited a similar trend to that observed in sWAT (*P* < 0.05, Fig. 4c–d and j–l). Consistently, immunohistochemistry demonstrated that UCP1 protein expression in sWAT was significantly lower in the HFD group than in the STD group (*P* < 0.05) and then significantly increased by CR (*P* < 0.05) (Fig. 4e, f).

### CR modulated the protein expression of NF-κB and eNOS

**Fig. 5:**
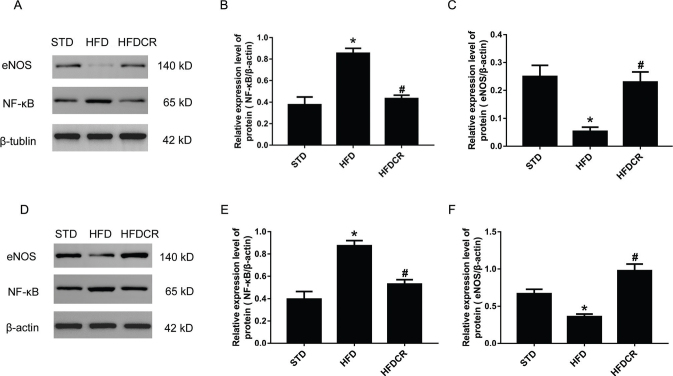
CR modulated the protein expression of NF-κB and eNOS. (a–c) Protein expression levels of nuclear factor kappa B (NF-κB) and endothelial nitric oxide synthase (eNOS) in sWAT, (d–f) protein expression levels of NF-κB and eNOS in hepatic tissue. Data are represented as mean ± standard error of the mean for *n* = 3 per group (*P < 0.05 vs*.* STD group and ^#^*P* < 0.05 vs*.* HFD group). Additional abbreviations: STD, standard diet; HFD, high-fat diet; HFDCR, high-fat diet with calorie restriction.

Compared with the STD group, the protein expression of NF-κB in sWAT was significantly higher in the HFD group (*P* < 0.05), but was inhibited by CR (*P* < 0.05, Fig. 5a, b). Furthermore, the expression of NF-κB in hepatic tissue exhibited a similar trend to that observed in sWAT (*P* < 0.05, Fig. 5d, e). The protein expression of eNOS in the liver and sWAT was significantly lower in the HFD group compared with the STD group, which was reversed by CR (*P* < 0.05, Fig. 5c and f).

### CR inhibited HFD-induced obesity via activating the AMPK/SIRT1 pathway

**Fig. 6:**
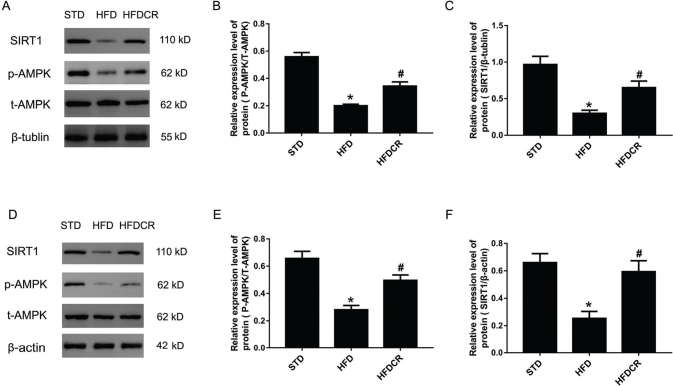
CR mediated AMPK/SIRT1 activity in subcutaneous white adipose tissue (sWAT) and hepatic tissue. (a–c) The expression levels of NAD-dependent deacetylase sirtuin-1 (SIRT1), phospho-AMP-activated protein kinase α (p-AMPKα), total-AMP-activated protein kinase α (t-AMPKα) proteins in sWAT, and (d–f) the expression levels of SIRT1, p-AMPK, and t-AMPK proteins in hepatic tissue. Data are represented as mean ± standard error of the mean for *n* = 3 per group (*P < 0.05 vs*.* STD group and ^#^*P* < 0.05 vs*.* HFD group). Additional abbreviations: STD, standard diet; HFD, high-fat diet; HFDCR, high-fat diet with calorie restriction.

The expression levels of p-AMPKα and SIRT1 in sWAT and hepatic tissue were significantly lower in the HFD group than in the STD group (*P* < 0.05), whereas significantly increased by CR (*P* < 0.05) (Fig. 6a–f).

## Discussion

The present study showed that CR decreased the body weight gain; the levels of serum TG, TC, and IR; the size of adipocytes; and the deposition of hepatic fat in mice with HFD-induced obesity. Moreover, CR enhanced heat production and oxygen consumption, indicating the elevation in the basal metabolic rate. At the molecular level, we found that the transcription of thermogenesis-related genes *UCP1* and *PGC-1α* in sWAT, and *UCP2* and *PGC-1α* in hepatic tissue was enhanced by CR, indicating that the CR induced weight loss and improved metabolism by activating thermogenesis. Moreover, we found that CR enhanced the phosphorylation of AMPK and the expression of SIRT1 in both sWAT and hepatic tissue. In addition, CR reduced the protein levels of NF-κB and increased eNOS protein expression, indicating CR inhibited inflammation. Taken together, CR can improve metabolic disorders induced by HFD through activating thermogenesis and reducing inflammation in sWAT and hepatic tissue by AMPK/SIRT1 signaling pathway.

In this study, we found that CR induced weight loss and improved glucose and lipid metabolism in HFD-induced obese mice. Thus, early CR intervention may defend against obesity. Compared with the HFD group, the daily intake of CR mice fed with a HFD was reduced by 25%. Without changing the composition of the diet, short-term CR was obviously effective in reducing weight gain in obese mice. However, the mechanism by which CR controls obesity is not fully understood. Obesity often arises from excessive food intake and/or low energy expenditure. Surplus energy is deposited in the form of adipose tissue in the liver, heart, and other organs. CR is the first choice to control obesity by decreasing energy intake and increasing energy expenditure in metabolic organs. We further investigated whether CR could stem white fat formation by activating thermogenesis.

Our study demonstrated that the low energy expenditure in HFD-induced obesity could be reversed by CR. Compared with the STD, the HFD increased the size of adipocytes in sWAT tissue, which was decreased by CR. In addition, CR significantly reduced the abdominal WAT volume in obese mice and restimulated heat production and oxygen consumption that had been significantly constrained by the HFD. However, CR did not modify the RER, which was significantly decreased by the HFD. The similar RER in HFD and HFDCR mice may be related to the same dietetic composition. Hence, CR significantly mitigated HFD-induced body weight gain and fat accumulation by boosting energy expenditure and heat generation in mice.

Moreover, CR increased the transcription of thermogenesis-related genes: *UCP1* and *PGC-1α* in sWAT, and *UCP2* and *PGC-1α* in hepatic tissue. Immunohistochemistry showed that the protein level of UCP1 in sWAT was increased in CR mice. Adipose tissue serves as a regulator in energy balance and nutritional homeostasis. BAT can function as an organ, which dissipate chemical energy as heat, and its thermogenic activity can combat obesity and diabetes (16). Moreover, UCP1 is an important BAT marker, and the ability of this tissue to dissipate energy is dependent on the expression of UCP1 (17, 18). Excess energy is stored as TGs in WAT, which can be converted into BAT-like beige fat tissue under hypothermia (17). We found that the mRNA and protein levels of UCP1 were increased in sWAT after CR treatment, indicating that WAT may be converted into beige fat tissue.

UCP1 and UCP2, which share a 59% structural similarity, play various roles in cellular metabolism, including glucose and lipid metabolism. UCP2 is found in mitochondria within adipose, hepatic, pulmonary, skeletal, and muscle tissue (18). In this study, CR increased the mRNA and protein levels of *UCP2* in hepatic tissue. BAT serves as a major site of non-shivering thermogenesis, while other tissues, hepatic in particular, can contribute to heat generation in cold-exposed conditions (11). Moreover, CR significantly rescued *PGC-1α* mRNA transcription and protein expression in sWAT and hepatic tissue of obese mice. PGC-1α is a master transcriptional regulator of mitochondrial remodeling and biogenesis. As a coactivator, it stimulates *UCP1* and *UCP2* expressions via many transcription factors, including members of the PPAR and C/EBP families (19, 20). Therefore, it is possible that CR may improve the basal metabolic rate and reduce fat accumulation by activating thermogenesis through the PGC-1α signaling pathway.

In this study, CR promoted the phosphorylation of AMPK in both sWAT and hepatic tissue in the HFD-induced obese mouse model. As the major sensor maintaining cellular energy homeostasis, AMPK is highly conserved across mammalian species (21). AMPK is expressed in hepatic, adipose tissue, and several key hypothalamic nuclei (6). It was reported that AMPK activation can reduce hepatic fat accumulation and enhance energy expenditure, lipid oxidation, and thermogenic capacity (22). Additionally, AMPK participates in the genesis of brown and beige adipose tissue. Pharmacological activation of AMPK induces WAT browning and increases energy expenditure (23). Moreover, AMPK regulates the expression of PGC-1α and mitochondrial proteins in mouse WAT (24). Thus, we speculate that CR-mediated activation of AMPK increases energy expenditure by regulating the expression of PGC-1α.

Our data showed that the expression of the SIRT1 protein in the CR group was elevated, compared to that in the HFD group. SIRT1 is a well-characterized metabolic modulator, which is activated to ameliorate HFD-induced obesity (25). SIRT1^−/−^ animals exhibited increased fat mass, impaired glucose tolerance, and attenuated insulin sensitivity (26). Therefore, CR may promote weight loss and improves obesity-related metabolic indices by activating SIRT1. A study showed that SIRT1 regulated the activity of PGC-1α in energy metabolism of different tissues (27). In our study, the expressions of transcription factors PGC-1α and SIRT1 were both increased in the obese mice after CR intervention, suggesting that CR boosted energy metabolism through the SIRT1 and PGC-1α signaling pathways. AMPK also indirectly regulates PGC-1α by stimulating the activity of SIRT1 (28). It was reported that CR gears the SIRT1/AMPK/PGC-1α pathway in mouse myocardium (29). The deficiency of skeletal muscle-specific AMPK reduces the beneficial effects of long-term CR, as SIRT1 and PGC-1α expressions are down-regulated (30). In this study, we found for the first time that short-term CR activates the AMPK/SIRT1/PGC-1α energy-sensing network in hepatic tissue and sWAT.

AMPK and SIRT1 are long-term partners (31). They have similar effects on diverse processes, such as cellular fuel metabolism, inflammation, and mitochondrial function. AMPK regulates energy expenditure by modulating NAD+ metabolism and SIRT1 activity, resulting in the deacetylation and modulation of the activity of downstream SIRT1 targets, namely, PGC-1α and the forkhead transcription factors (FOXO) FOXO1 and FOXO3a (32). Moreover, SIRT1 deacetylates the targets of the LKB1 kinase and then phosphorylates AMPK, which further drives its activation (33). AMPK and SIRT1 regulate each other and share many common target molecules, including PGC-1α, eNOS, NF-κB, and FOXO (33).

Obesity is a low-grade sustained inflammatory state that causes oxidative stress in different metabolic tissues, which leads to IR and non-alcoholic fatty liver disease (NAFLD) (34, 35). Abnormal activation of NF-κB has been implicated in many pathological conditions such as inflammation. In the present study, we confirmed that CR inhibited the HFD-induced activation of NF-κB signaling in the liver and WAT. A recent study revealed an important molecular role for hepatocyte-specific eNOS as a key target for NAFLD/non-alcoholic steatohepatitis (NASH) susceptibility, mitochondrial biogenesis, and inflammation attenuation (36, 37). Moreover, eNOS plays an important role in adiponectin synthesis in adipocytes by increasing mitochondrial biogenesis and enhancing mitochondrial function (38). It was found that obesity reduces the eNOS level in adipose tissue (39). In this study, we found that HFD-induced obesity reduces eNOS expression in both liver and adipose tissue, while CR increases its expression, which may be related to inflammation inhibition and mitochondrial function improvement.

In conclusion, CR may ameliorate obesity and associated metabolic disorders by activating the AMPK/SIRT1/PGC-1α energy-sensing network and mitochondria-mediated thermogenesis. Moreover, CR may alleviate obesity-related metabolic abnormalities by inhibiting inflammation in liver and adipose tissue. However, this hypothesis should be validated by future studies, investigating whether the inhibition of SIRT1 or AMPK abrogates the effects of CR on WAT browning and hepatic lipid metabolism.
